# Editorial: Brain stimulation methods in human motor neuroscience

**DOI:** 10.3389/fnhum.2024.1532408

**Published:** 2024-12-20

**Authors:** Syoichi Tashiro

**Affiliations:** ^1^Department of Rehabilitation Medicine, Faculty of Medicine, Kyorin University, Mitaka, Japan; ^2^Department of Rehabilitation Medicine, School of Medicine, Keio University, Shinjuku, Japan

**Keywords:** aphasia, hemispatial neglect, neuromuscular electrical stimulation, neurorehabilitation, non-invasive transcranial brain stimulation, non-invasive transcranial focal electrical stimulation, stroke, vision disorders

Stroke is a leading cause of disability and induces an enormous burden of care as well as economic loss. While various neurorehabilitation methods, including those utilizing physical modalities, are used, the degree of recovery is not favorable in the chronic phase. Non-invasive transcranial brain stimulation (NTBS) has attracted wide attention as it modifies brain state and plasticity via electrical or magnetic stimulation of the brain presenting functional impairment. Transcranial electric current stimulation has the advantages of simplicity and higher practicality than magnetic stimulation. Although transcranial direct current stimulation (tDCS) has been investigated for decades, its clinical application has been limited by variability in efficacy. On the other hand, investigations on transcranial alternating current stimulation (tACS) have only recently begun. It has been suggested that tACS exerts its effect via the entrainment phenomenon to synchronize the brain's endogenous rhythm with the frequency of the exogenous AC current applied transcranially. However, it is only recently that tACS studies were initiated in stroke patients. This editorial introduces the current status of the clinical application of tACS in stroke treatment.

Only two studies have been published on the treatment of hemiparesis. Wu et al. (2016) applied 400 μA beta-tACS for 30 min to the bilateral mastoid of patients with subacute stroke in an RCT. The NIH Stroke score of the active stimulation group was significantly decreased, accompanied by better hemodynamic parameters, after 15 intervention sessions. Kitatani et al. (2020) applied 2.0 mA beta-tACS over the primary motor cortex (M1) during 10 min of treadmill training twice weekly for 5 weeks and investigated the aftereffect on EMG-EMG coherence in chronic stroke patients. This pilot study combined neuromuscular electrical stimulation (NMES) targeting the tibialis anterior (TA) muscle. Beta-band coherence of the paretic TA muscle increased during the overground gait evaluation without tACS. In addition, the change in beta-band coherence was correlated with gait distance within 6 min. In addition, some research groups have published trial protocols for tACS applications that target motor recovery. Lai et al. (2024) focused on the specific effects of multitarget tACS at the gamma frequency. They will convey 20-min, 1.0 mA tACS daily over 2 weeks in parallel with rehabilitation training. Tashiro et al. (2024) intend to apply focal intermittent individualized beta-tACS targeting the specific timing of repetitive finger extension training to restore beta rebound in chronic stroke patients. Their approach included the application of closed-loop NMES to assist peripheral muscle contraction and enhance the treatment effect.

Because stroke induces various higher-level cognitive impairments, some researchers have focused on representative symptoms as targets of tACS. Three studies were conducted in total. Xie et al. (2022) applied theta tACS to target aphasia in patients with chronic stroke. High-definition tACS targeting supplementary motor area was applied in the first 30 min of a 2-h speech-language therapy (SLT) session in a 2-week intervention. They demonstrated that active tACS strengthened the efficacy of SLT and improved speech comprehension. Following a within-subject, placebo-controlled study by (Schuhmann et al., 2022; Middag-van Spanje et al., 2024) applied tACS to treat hemispatial neglect of chronic patients in an RCT. A focal alpha-tACS was applied to the contralesional parietal cortex at 1.5 mA with 40 min of visual scanning training. After 18 training sessions over 6 weeks, significant improvements in visual search and detection were induced in the active stimulation group despite no effect on the line bisection test and activity of daily living (ADL) index.

Targeting other symptoms of stroke, Xu et al. (2021) studied visual field restoration secondary to tACS-tDCS and assessed functional connectivity using functional MRI in patients with occipital stroke. Following O1/2-Fpz tDCS at 1.0 mA, tACS was applied using an Fpz-upper arm montage at frequencies incremented from 1 to 30 Hz at 1.5 mA for 20 min. While there were responders and non-responders, the combinatorial treatment enhanced functional connectivity between the occipital and temporal lobes in the intact hemisphere and decreased low-frequency coherence between the damaged and intact occipital brain areas in responders.

In addition to the treatment mentioned above, some researchers have studied the effect of tACS on stroke patients from more experimental aspects. Naros and Gharabaghi (2017) investigated the effect of conditioning with 20 Hz tACS at 1.1 mA targeting the lesioned motor cortex. They compared two conditions, in which tACS was applied continuously before or intermittently during the motor imagery task. They found that intermittent tACS improved the classification accuracy by reducing the variance of resting oscillations. Chen et al. (2021) compared the effects of 10 Hz and 20 Hz applied at 1.0 mA via an M1-supraorbital montage. While local segregation in motor-related regions was facilitated by 10 Hz tACS, the nodal clustering characteristics decreased after 10 Hz and increased after 20 Hz. Bevilacqua et al. (2024) applied alpha-gamma bifocal tACS targeting primary visual cortex (V1) and the medio-temporal area (MT) to modulate the cortical visual motion network in chronic stroke patients with occipital lesions in a crossover design: forward tACS (V1alpha-MTgamma) first or backward tACS (V1gamma-MTalpha) first. They found that single-session bifocal forward-tACS increased bottom-up connectivity, as assessed by phase-amplitude coupling. Grigutsch et al. (2024) applied theta-gamma peak-coupled tACS with the aim of motor skill acquisition in patients with chronic stroke. A high-density tACS at 2.0 mA peak-to-baseline intensity was applied on the M1 of the affected side during six blocks of an acceleration-dependent thumb movement task over 38 min in total. However, active stimulation diminished motor skill acquisition compared with sham stimulation. Omae et al. (2024) targeting post-stroke aphasia applied 15 Hz amplitude-modulated tACS between Broca's and the right homotopic areas with increased inter-regional synchrony through several sessions.

Several researchers have introduced the tACS in clinical settings to target stroke patients, but the target symptoms and stimulation conditions are still highly variable, and the reported effects are still not remarkable. It is noteworthy that some research group combines the other electrical stimulation to the periphery to support voluntary motor output. While some studies have used amplitude-modulated tACS comprising carrier and modulation frequencies, its physiological effects should also be clarified. Therefore, further studies are warranted.
